# Short-term cognitive learning outcomes in team-based learning: is the permanent team important?

**DOI:** 10.1080/10872981.2024.2397864

**Published:** 2024-09-05

**Authors:** Stefan Heber, Michaela Wagner-Menghin, Ivo Volf, Marjan Slak Rupnik, Diethart Schmid, Richard Marz, Michael J.M. Fischer

**Affiliations:** a Center for Physiology and Pharmacology, Medical University of Vienna, Vienna, Austria; b Department of Psychiatry and Psychotherapy, Clinical Division for Social Psychiatry, Medical University of Vienna, Vienna, Austria; c Medical University of Vienna International, Vienna, Austria

**Keywords:** Team-based learning, online teaching, collaborative learning, group composition, temporary team

## Abstract

Assigning students to work in permanent teams is a design principle in Team-based learning (TBL). It has been assumed that a stable team composition supports the emergence of collaborative problem-solving and learning: when students became more familiar with each other, they shared more information and resolved discrepancies together, which in turn stimulated knowledge acquisition and comprehension. However, this assumption had not been probed by a randomized controlled trial with performance assessment as an outcome. In an online course for second term medical students, 50% of the students were reassigned to new teams for each of the 24 problems to be solved during four classes, thus precluding familiarity. The learning outcome was assessed shortly after the third of four classes by a domain knowledge test. Whether TBL teams were permanent or temporary did not affect the score of a domain knowledge test. As expected, participation in online TBL improved the domain knowledge test results. Overall, the permanent team seems to be less important for cognitive learning outcomes than previously assumed, but this may depend on the specific educational setting. However, team familiarity may still be important for team decision-making. As clinical reasoning in the medical workplace often involves collaborating in changing teams, future research on TBL should focus on how to utilize this format to prepare medical students for decision-making and optimal learning outcomes under these conditions.

## Introduction

Team-based learning (TBL) has been introduced as a format for collaborative learning for large groups [1,2]. Originally used for face-to-face classroom settings, the TBL-format also proved effective in virtual environments [3]. The TBL format comprises separate phases to actively engage students in their learning: First, students are expected to prepare on their own to acquire domain knowledge necessary to engage in the TBL. At the start of each TBL session, students’ individual readiness to solve application-oriented problems is assessed, which is referred to as ‘individual Readiness Assurance Test’ (iRAT). Subsequently, teams of 5–7 students discuss the same problems and have to make a team decision. This is referred to as ‘team Readiness Assurance Test’ (tRAT). These decisions are then argued and defended in discussion, moderated by the instructor, who also provides feedback. Additionally, problems that are more complex can be presented as part of the session.

TBL has been demonstrated to improve interpersonal skills and problem solving skills and to encourage deep learning [4–6]. It was suggested that effective TBL requires problems whose decisions can be reported in a simple form and engage students in a content related discussion, rather than problems requiring the generation of lengthy output. Additionally, it has been reported that crucial factors for the successful implementation of TBL include providing students with frequent and timely feedback, and forming strategically permanent teams [7]. It was recommended to allocate intellectual assets and potentially different viewpoints evenly across all teams. The foundational practice to have permanent TBL teams [7], working together over an extended period for effective knowledge acquisition, is based on group dynamic research, which suggests that teams need time to learn to collaborate effectively. It has been reported that the process by which tRAT solutions were arrived at was developed throughout a term [8]. On their first and second tRAT, approximately two-thirds of undergraduate teams missed out on tRAT credits, because they did not agree on a single group solution, which, when correct, would have earned them full credit. Rather, they split their risk among different solution possibilities, of which only one could be correct. This behaviour had fallen to 10% by the fourth to sixth tRATs. Team familiarity and trust developed as students repeatedly engaged in solving problems together, thus enabling them to collaboratively solve the problem. Five rounds of problem-solving as a team were sufficient for that switch. However, the assumption that permanent teams foster knowledge acquisition and comprehension in TBL has not been tested by a randomized controlled trial, which is regarded as the gold standard for investigating cause-and-effect relationships [9]. The present study aims to fill this gap by a two-arm study manipulating team familiarity to compare the cognitive learning outcome of permanent teams (arm 1) vs. temporary teams (arm 2).

One can construct a competing hypothesis, based on results from studies on the performance of clinical teams solving diagnostic problems [10]. A review of the factors influencing diagnostic reasoning performance in teams concludes that the ‘distribution of information plays an integral role throughout the reasoning process’ [11]. Within clinical teams, observations shared by more experienced team members were valued more compared to observations made by less experienced members. In clinical teams with members of similar level of expertise, diagnostic accuracy might be better, because the reciprocally perceived similarity in expertise increases the sharing and the uptake of information [12]. Similarly, with increasing familiarity in permanent TBL teams, members can appraise each other’s knowledge and establish an expertise hierarchy. Perceived differences in expertise might thus negatively influence the collaboration process and its outcome. In contrast, in temporary teams with less or no familiarity, appraising each other’s level of expertise is more difficult or not possible. All contributions would then be acknowledged open-mindedly and equally, which could motivate information sharing and in-depth discussion.

The SARS-CoV-2 pandemic-induced need to transform face-to-face to online sessions provided an opportunity to conduct an experiment: Does team familiarity influence the acquisition of domain knowledge? Managing the TBL teams within the video conferencing tool used for online TBL sessions allowed control of team familiarity by assigning students either to a ‘permanent’ team (working together the whole course) or a ‘temporary’ team (working together for only one problem). This frequency of reassignment is impractical to manage in a face-to-face session. Specifically, the study addresses the following question: How does working as a member of a permanent or a temporary team during a TBL session influence a student’s cognitive learning outcome, measured by a domain knowledge test?

## Methods

### Setting

**Figure 1.**
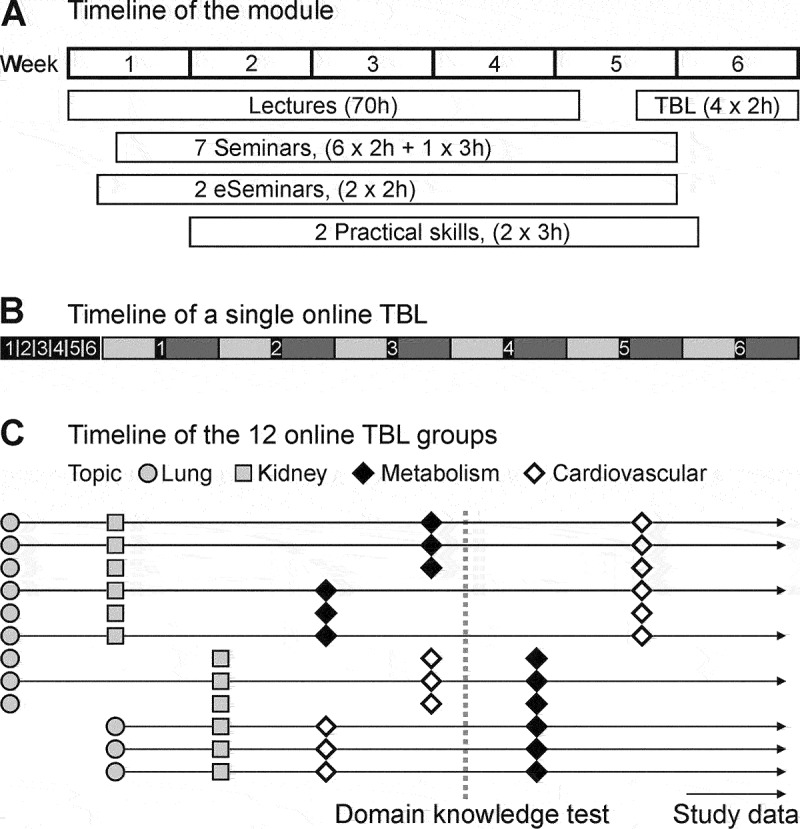
A) Timeline of the module, including the four online Team-based learning (TBL) sessions within the last two weeks. B) Timeline of a single online TBL session (90 min). Six consecutive iRATs are followed by six discussion-decision-feedback cycles. Each problem is discussed in the team (light grey), the group decision is communicated (tRAT, black with white numbers) and feedback is provided by the teacher in the plenary discussion (dark grey). C) Topics of the online TBLs for all 12 sets of 60–70 students. Note the different orders for the final two topics. The dotted line indicates the domain knowledge test after the third online TBL.

The study population were second semester medical students at the Medical University of Vienna. At the time of the study, the students had a median age of 20.2 years. The study was implemented in the TBL sessions of the module ‘Functional Systems and Biological Regulation’, which is located at the beginning of the second semester. The 6-week module consists of 70 h of lectures and 25 h of small group seminars and practical skills training, which build on the knowledge acquired in the first term. Lectures, seminars, and practical skills training in weeks 1–4 prepared for the four compulsory TBL sessions (Figure 1A). Each TBL session covered a separate topic. The 740 first-year students were placed in 12 parallel batches of students (10 with 60 students and 2 with 70 students) for the TBL sessions to allow a meaningful plenary discussion.

### Team composition

Each semester students register for one of 74 ‘groups’ of 10 students. All small-group activities, such as seminars and practical courses, are attended as a permanent group. Thus, the students spent at least 25 h working together prior to entering their first TBL-session and had time to become rather familiar with each other. The intervention of this study was to either utilise or preclude the development of team familiarity during TBL sessions for half the students. Within each ‘group’, five students were randomly assigned to a permanent team, and five to temporary teams consisting of alternating members of different groups. In this randomised controlled study design, observed differences in domain knowledge Test (dkTest) scores between the groups can be interpreted as causal effects of team-familiarity on learning outcomes. To maximise the non-familiarity, students from five different groups were assigned to temporary teams based on a 6 × 6 Latin squares design (Online Resource Fig. S1). There was little familiarity stemming from the first term; among all 6 temporary teams in one TBL session, on average less than one other student was from the same ‘group’ of the first term.

### Readiness assurance procedure

Each of the online TBL sessions included six problems, each presented as a question with five true or false answers, which allows simple reporting. First, students had to complete the six problems individually without communicating with each other (=iRAT, Figure 1B). Subsequently, teams discussed the first problem in breakout rooms. Back in the plenary room, each student completed the true or false items again by entering the group decision (=tRAT). Next, the lecturer inquired about the rationale for each answer, clarified misconceptions, and provided relevant details. The other five problems were completed in an identical fashion (Figure 1C). It has been published that TBL allows students to work on complex problems [2], which is also reflected in our problems, with several items designed to be partially ambiguous to challenge students and provoke discussion. To comply with a suggested reporting standard for TBL [13] additional details are provided regarding the TBL procedure not mentioned otherwise (Online Resource Table S1).

### Implementation of online TBL

The online TBL sessions were implemented using the video conferencing platform Webex. During the first year of the SARS-CoV-2 pandemic, the teaching faculty and the students had gained routine in using the video conferencing platform of the university, starting summer term 2020. The data presented here were acquired in March–April 2021. Students used the learning management system Moodle, which supports teaching at the university since 15 years.

### Teachers’ perspective

Six teachers were chosen to teach online TBL, each teacher facilitated two batches of students. Training included a presentation with screenshots distributed 6 weeks ahead of time. In a 2-h online training the day before the first online TBL, all teachers practised how to manage breakout rooms, operate the polling tool, and handle results without additional personnel support. A single page summarising the procedure (Online Resource Fig. S2) and the problems’ solutions were also provided. A separate *.txt file was generated for every problem. Teachers loaded these into the conference room using the polling tool, once for the iRAT and again after group discussion for the tRAT. Anonymized tRAT results were reflected back to the students for the plenary discussion.

Teachers were invited 8 days before the first online TBL to participate in the study. They were informed about the requirements of participation: save the results of the iRATs and tRATs and administer a questionnaire at the end of the last online TBL also using the polling tool. Four of six teachers agreed to participate in the study and provided the respective data. These teachers had 3–11 years of experience with TBL in lecture halls. Course content was identical regardless of teacher.

### Student’s perspective

Students were provided with detailed instructions for the online TBLs. These included that each TBL session focuses on one topic and includes six problems, each presented with five true or false answers. Instructions included the timeline of 1.5 min for providing an individual answer, 5 min of discussion of the topic in a breakout room in a team of five students, a group vote after return to the main session within 1 min, and an interactive discussion of all answers with direct teacher feedback, lasting about 5–7 min. Students were also instructed about the different teams and the purpose to optimise future online TBLs. The importance of interaction within one’s team for maximum learning outcome was stressed. It was recommended to activate the camera, and due to the polls and breakout sessions, to use the desktop application of the video conferencing software on a computer. Students were informed that the software recorded the answers. As this was the students’ first encounter with TBL, its concept was explained. A reference to the English team-based learning Wikipedia article was provided. To discourage students from obtaining problems and answers from students of other batches, course credits were earned based on attendance only. It was emphasised that looking up the correct answer in repositories would limit their learning opportunities.

### Study design

As a primary endpoint, a randomised controlled trial investigated whether team familiarity in TBL affects domain knowledge acquisition. The latter was assessed by a mandatory domain knowledge test, designed to assess understanding in distinct areas. As a reference for the primary endpoint, it was examined whether online TBL, independent of team familiarity, improved domain knowledge acquisition. All students worked on the same topics for the first and second TBL session. In the third online TBL session, one batch per teacher worked on ‘metabolism’ followed by ‘cardiovascular’, while for the other batch the order was reversed. The dkTest between the third and the fourth online TBL allowed a direct evaluation of the effect of participating in a TBL session on knowledge acquisition in a specific domain.

### Primary outcome: domain knowledge

The dkTest used 12 test-problems, similar to the problems used for the TBL sessions, but applied as a speed and power test via the Learning Management System. The dkTest covered topics from the preceding lectures, seminars, and TBLs. Problems were presented as questions together with their five true or false answers in individually randomised order. There were no restrictions on where to take the test, but the date and time for taking the test was fixed. The time limit for the test was 18 min, to be completed within a 19-min time-slot. There was no going back to a problem after it had been submitted. Students were informed in detail about the test format. The primary outcome variable dkTest was derived as the sum of correctly decided true or false items. Each topic’s domain knowledge was assessed with 15 true or false statements, resulting in a domain knowledge score of 0–15 for both ‘metabolism’ and ‘cardiovascular’.

### Effect of team composition on dkTest score

To assess the primary endpoint, a linear mixed model approach was applied. The predictors team composition (permanent vs. temporary), dkTest-topic (Metabolism vs. Cardiovascular), and TBL-topic covered prior to dkTest (Metabolism vs. Cardiovascular) were entered as fixed factors in a full factorial model, i.e., with all main effects, the three possible two-way interactions, and the three-way interaction. To account for the reduced variance due to clustered data (e.g., students taught by the same teacher), the factors ‘student’, ‘group’, and ‘teacher’ were included in the model; ‘student’ and ‘group’ were used as random factors. As there were only four teachers, ‘teacher’ was included as an additional fixed factor, yet without any interaction terms with the other fixed effects. The two-way interaction between team composition and TBL-topic covered prior to the dkTest represented the test for the primary endpoint. Using contrasts, the dkTest scores of permanent and temporary teams were compared. Least squares means derived from the full factorial model and contrasts estimates are depicted together with raw data.

To explore effects of sex, the binary factor ‘Sex’ was included in the model with all possible interactions. A total of 490 students were scheduled to attend the sessions, data were acquired for 464 students, of which 56% were female. IBM SPSS Statistics 29, Module ‘MIXED’ was used for the primary analysis. Approximate normal distribution of residuals was inspected visually. Two-sided P-values were used, *p* ≤ 0.05 was considered statistically significant.

### Effect of online TBL on dkTest scores

As reference, it was assessed whether and how much participation in online TBL improved the topic-specific dkTest scores. To this end, the mean test scores were compared between students who already attended the relevant TBL to those who did not. A mixed model was used to test whether this was different for ‘cardiovascular’ and ‘metabolism’. Within the topics, the results of students who had attended the topic were compared to those who had not with an independent-samples t-test.

### Data protection, confidentiality, and privacy

Study design and data handling have been approved by the Data Protection Committee and by the ‘Clearing Stelle Lehre’ of the Medical University of Vienna.

## Results

### Effects of team composition on domain knowledge test score

**Figure 2.**
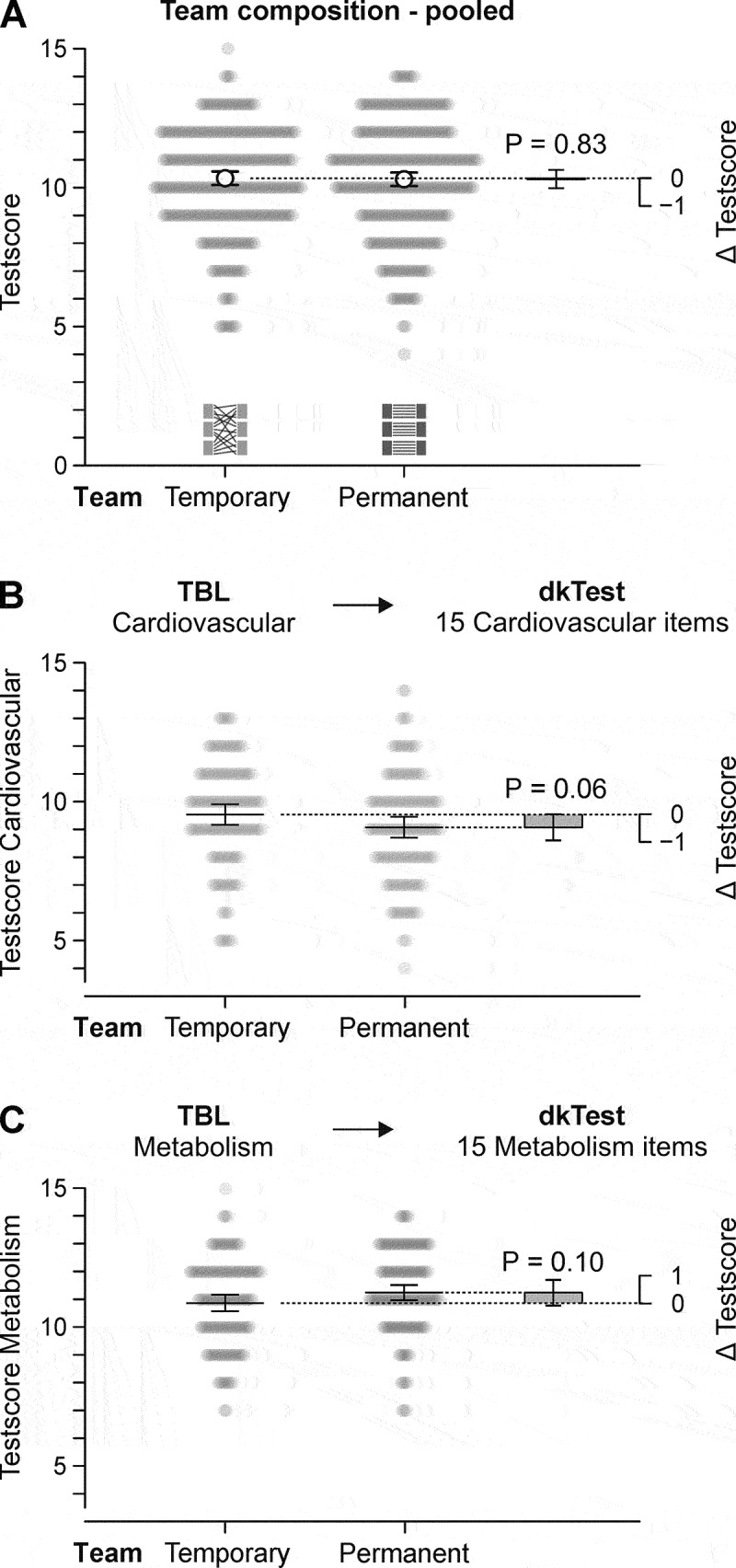
Team composition does not affect domain knowledge test scores. A) Comparison of domain knowledge test (dkTest) scores pooled for both topics between permanent and temporary teams who worked on the matching topic in the TBL preceding the dkTest. B) Comparison of dkTest scores regarding ‘cardiovascular’ between students of permanent and temporary teams who solved cardiovascular problems during the TBL preceding the domain knowledge test. C) Comparison of dkTest scores regarding ‘metabolism’ between students of permanent and temporary teams who solved metabolism problems during the TBL preceding the dkTest. Grey symbols represent the score of a student. Other symbols in all panels represent least squares arithmetic means or estimated mean differences. All error bars are 95% confidence intervals.

The hypothesis that students working in permanent teams would benefit more from online TBL sessions than students working in temporary teams was investigated. However, there was no evidence that team composition affects domain knowledge acquisition. Students working in permanent teams had nearly identical dkTest scores as those working in temporary teams (estimated dkTest score difference = 0.034 points, 95% confidence interval −0.29–0.36, Figure 2A). Due to the study design, the analysis mentioned above included two subgroups of students. For the subgroup which attended the ‘cardiovascular’ TBL prior to their dkTest, the dkTest items covering the topic ‘cardiovascular’ were included in the analysis, and dkTest items on the topic ‘metabolism’ for those who participated in this TBL. Analysed separately, neither subgroup provided evidence that permanent teams benefited more from online TBL sessions than students working in temporary teams. For ‘cardiovascular’ TBL prior to the dkTest, students from permanent teams tended to perform worse than students from temporary teams in the respective topic (*p* = 0.06, Figure 2B). For ‘metabolism’ TBL prior to the dkTest, there was no evidence of a benefit of permanent teams (*p* = 0.10, Figure 2C).

The exploratory extension of the model by the factor ‘sex’ and its interactions did not suggest that the above-mentioned results are different by sex (all interaction terms with sex *p* > 0.15).

As negative controls, the dkTest scores of questions referring to the TBL topic students did not attend prior to their dkTest were analysed. For those who had the ‘cardiovascular’ TBL prior to their dkTest, the dkTest questions referring to ‘metabolism’ were analysed, and vice versa. As expected, whether the students were part of permanent or temporary teams did not affect the test scores (*p* > 0.45 each).

### Effect of TBL on domain knowledge test scores

**Figure 3.**
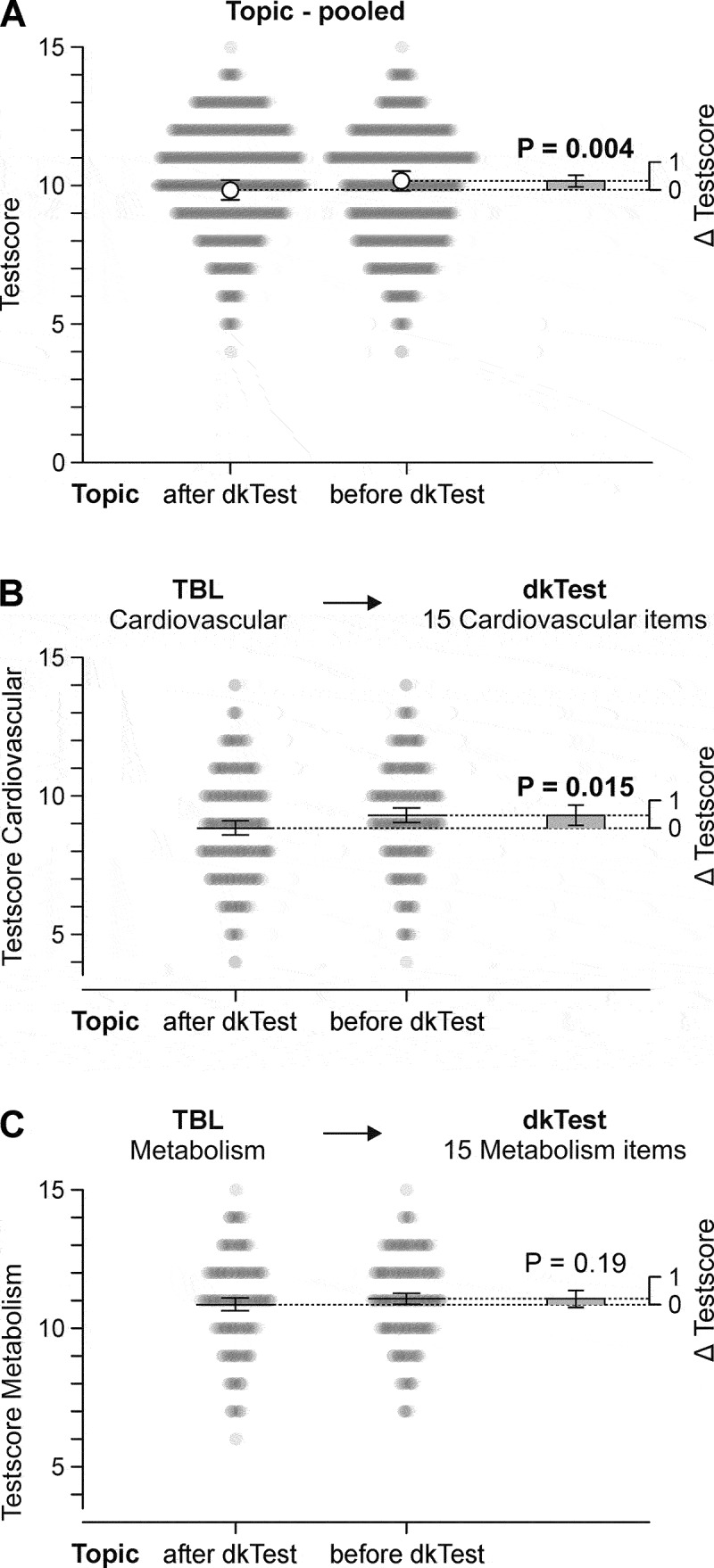
Online TBL improves topic-specific test scores. A) Each semi-transparent symbol represents the score of one of 464 students. Least squares means resulting from a mixed linear model estimating the main effect of having a topicwise appropriate online TBL session before the domain knowledge test. The bar represents the estimated difference. All error bars are 95% confidence intervals. B) Subgroup analysis of scores relating to the ‘cardiovascular’ TBL. C) Subgroup analysis of scores relating to ‘metabolism’ TBL. The domain knowledge test contained 60 items, 15 related to ‘cardiovascular’, 15 to ‘metabolism’, and 30 to other topics.

The next step was to investigate whether TBL in an online setting, regardless of whether permanent or temporary teams were used, improved the dkTest scores. The applied mixed linear model did not indicate a difference between the effects of ‘cardiovascular’ and ‘metabolism’ (*p* = 0.34), therefore the data were pooled for analysis. Having a topic in an online TBL session before the dkTest was associated with a higher score by 0.33 points (0.10–0.55, main effect ‘taught or not’ *p* = 0.004, least squares means, Figure 3A). In relative terms, this corresponds to a 3.3% improvement on the dkTest that is associated with an online TBL session with the appropriate topic compared to the other topic. In a separated analysis for the two subgroups, students who had an online TBL session with cardiovascular as topic prior to their dkTest achieved 0.45 points (95% CI 0.09–0.81) more on cardiovascular-related dkTest problems than students who had online TBL metabolism before the dkTest (Figure 3B). The mean score of those students with metabolism as online TBL topic prior to their dkTest was 0.21 points (95% CI −0.10–0.51) above on metabolism-related dkTest problems compared to students who had online TBL cardiovascular before the dkTest (Figure 3C). The dkTest scores referring to the other TBL-topics ‘lung’ and ‘kidney’ were used as negative control. Overall, there was no difference in the sum of the other 30 binary scores between those who had metabolism and those who had cardiovascular in the third online TBL (*p* = 0.38). There was no evidence of any sex-effect in these analyses (all interaction terms with sex *p* > 0.67).

## Discussion

The necessity for permanent teams as a foundational design principle in TBL was investigated by manipulating team familiarity. Surprisingly, team familiarity did not affect acquisition of domain knowledge by medical students. Participating in TBL improved domain knowledge of this specific topic while participating in TBL for a different topic did not.

### Team composition

The two-arm experimental study investigated the importance of permanent teams for individual learning outcome, measured by a domain knowledge test, without assuming that temporary teams were better. The upper 95% CI limit of the contrast comparing the learning outcome between permanent vs. temporary teams matched the difference observed in the domain knowledge test depending on the topic being covered in TBL. Therefore, team composition is likely to have little or no effect on learning outcome. However, the processes occurring within permanent and temporary teams might still be different. It appears plausible that there are tasks for which temporary teams perform better than permanent ones. In medical education, acquisition of domain knowledge is very important, and the results demonstrate that this is successful in temporary teams. The permanent teams of TBL do not reflect the clinical workplace, where clinical teams change frequently, due to rotations and requirements of individual duty schedules. However, temporary teams affect communication, coordination, and synergy among healthcare professionals. This might ultimately affect overall healthcare quality [11]. An argument in favor of temporary teams could be the reduction of counter-productive team dynamics. Another consideration concerns the development of effective decision-making skills. Medical training aims to prepare students for clinical decision-making, which should be effective, irrespective of team composition. Education in temporary teams might prepare for this clinical situation, and mimicking the latter should be considered when effectiveness of teaching does not indicate a fixed team composition. The highly structured context of TBL in the classroom provides the opportunity to further research factors influencing the effectiveness of collaborative decisions in temporary and permanent teams.

Learning outcomes probed by the dkTest and the RATs might be different. The dkTest is an index of depth of knowledge, while tRATs are at least also determined by problem-solving and communication skills. For the research question relevant to this study, the dkTest seemed better suited as an outcome variable than iRAT or tRAT.

Our implementation of TBL requires further explanation, as it might affect how well the results translate to another setting: The module lasts 6 weeks and includes four TBL-sessions in the final 2 weeks. At this time, basic domain knowledge is expected, and the main aim is critical discussion and clinical reasoning, supported by immediate feedback. The number of TBLs as well as the duration matches common practice [14]. There is no out-of-class group assignment, resulting in less time for group dynamics and team-building than in other settings. However, the members of the permanent teams are also members of a permanent group for all other small group activities of the semester, so they know each other well. This is in contrast to temporary teams. Students were informed that low RAT scores would not impact their grades. This decision was made to avoid any incentive for pre-acquiring correct answers, defeating the TBL concept of ‘complex reasoning, debate and constructive controversy’. However, the lack of such an incentive is likely to result in less team interaction and therefore team building. Nevertheless, TBL at the Medical University of Vienna has relied on the intrinsic motivational power of the method rather than on assigning grades on RATs since 2004 [15].

Previous studies controlled the diversity of prior domain knowledge, familiarity, and other learner characteristics and have suggested that ‘most of the reported “problems” with learning groups (free-riders, member conflict, etc.) are the direct result of inappropriate group assignments’ [16]. In the present study, students were randomly assigned into permanent and temporary teams. This prevented self-formed groups of friends, but other learner characteristics were left to chance.

The factor sex was considered in all analyses, as sex differences appeared a priori plausible. However, there was no sex differences in dkTest scores dependent on team composition. Also, the benefit of online TBL teaching on dkTest score improvement did not differ by sex.

### Online vs. in presence TBL

There are guidelines and recommendations for online TBL [17,18]. Many aspects of the process of translating to an online course have been reported, but the composition of the team has not been considered [18]. There are a few recent studies comparing face-to-face with online TBL sessions: A comparison of voluntary in-person and online TBL participation, likely to have a selection bias in those attending, found that in-person TBL attendees were 17% better than non-attendees, online TBL attendees were only 7% better than non-attendees [19]. This study did not mention team composition, not even whether this was self-chosen or randomized by the university. Another study focused on the feasibility of online TBL, without reporting results or comparing different experimental arms [20]. An interventional study of a single TBL session found that synchronous team-based learning increased test scores more than self-study [21]. Online TBLs were positively received compared with in presence settings across different study programs [3]. The study randomized students into permanent teams, and the decision was not elaborated on. Others have provided advice based on their experiences in written, but non-peer reviewed form: Compared to suggestions of having a ‘Web conference host’, a ‘TBL system coordinator’, a ‘Facilitator’, and a ‘Subject matter expert’ [22], our approach appears lean, managing everything with a single well-trained teacher.

TBL has been used with different class sizes, and the latter has not influenced learning outcomes [23]. The batch size of 60 students from pre-Covid19 times was maintained for online TBL, due to existing scheduling and to allow personal interaction. After each of the six tRATs, the five items and the elaborated discussion of the problem allowed the teacher to interact with the majority of students in every class.

### Limitations

There are multiple other outcomes than cognitive learning outcomes related to team-based learning, which were not the focus of the present study, but may well depend on team familiarity. However, a recent review shows that about 80% of TBL implementation studies in undergraduate medical education report cognitive learning outcomes such as knowledge acquisition and comprehension, followed by students’ satisfaction or attitudes toward TBL in about 70% [24].

The study addressed the effects of familiarity in teams where all team members had the same role in contributing to the team task on individual content domain knowledge as outcome. The study was not done on teams with formally assigned or professionally established roles (as e.g., in aviation or surgical teams).

The study was performed in an online setting, therefore it remains a (testable) hypothesis, whether these findings apply to the classroom setting.

While it has been possible to implement an experimental study in a real-life educational setting, providing high ecological validity of the experiment, there are several limitations related to conducting an experiment in a real-life setting. While the number of four TBL-sessions within a 2-week period may seem too short to establish familiarity between team members, students who were assigned to permanent TBL teams had already spent at least 25 h within 3 weeks working together prior to entering their first TBL-session.

## Conclusion

In the present study, medical students working in temporary or permanent teams benefit equally from TBL regarding short-term cognitive learning outcome measured by domain knowledge tests. This challenges the established position that permanent teams are fundamental to cognitive outcomes in the TBL setting. As such, the following practice points can be taken from the results:

1. For knowledge acquisition, a more relaxed approach can be applied to the formation of TBL learning groups. While students should not self-enroll, it seems less important to control team familiarity.
2. Team composition may influence outcomes beyond domain knowledge acquisition. However, this requires further investigation to allow for an evidence-based decision regarding team formation in this educational setting.
3. Students need effective collaboration skills when transitioning from classroom- to workplace-learning. Collaboration in temporary teams mirrors the working and learning conditions found in the clinical workplace; therefore, students are likely to benefit from practicing collaborating in temporary teams.

## Supplementary Material

Supplemental Material

## Data Availability

The data that support the findings of this study are available upon reasonable request. The data are not publicly available due to privacy or ethical restrictions.

## Abbreviations

dkTest: Domain knowledge test
RAT: Readiness assurance test
TBL: Team-based learning

## References

[1] Michaelsen LK, Watson WE, Black RH. A realistic test of individual versus group consensus decision making. J Appl Phychol. 1989;74(5):834–9. doi: 10.1037/0021-9010.74.5.834

[2] Michaelsen LK, Sweet M. The essential elements of team-based learning. New Dir Teach Learn. 2008;2008(116):7–27. doi: 10.1002/tl.330

[3] Silva ECE, Lino-Neto T, Ribeiro E, et al. Going virtual and going wide: comparing team-based learning in-class versus online and across disciplines. Educ Inf Technol. 2022;27(2):2311–2329. doi: 10.1007/s10639-021-10683-0 PMC8366158 34421327

[4] Kelly PA, Haidet P, Schneider V, et al. A comparison of in-class learner engagement across lecture, problem-based learning, and team learning using the STROBE classroom observation tool. Teach Learn Med. 2005;17(2):112–118. doi: 10.1207/s15328015tlm1702_4 15833720

[5] Fink LKM Arletta Bauman Knight, L Dee, editors. Team-based learning: a transformative use of small groups in college teaching. (NY): Routledge; 2023.

[6] Hai MA, Geraets JA. An assessment of students’ enthusiasm for pre-class preparation. Open J Soc Sci. 2023;11(08):112–134. doi: 10.4236/jss.2023.118008

[7] Michaelsen L, Davidson N, Major CH. Team-based learning practices and principles in comparison with cooperative learning and problem-based learning. J Excell Coll Teach. 2014;25:57–84. https://celt.miamioh.edu/ojs/index.php/JECT/article/view/466

[8] Birmingham C, Michaelsen L. Conflict resolution in decision making teams: a longitudinal study. Proceedings of the Midwest Academy of Management; August 6–11; Chicago, IL; 1999 [cited 2023 Dec 13]. https://www.researchgate.net/publication/255647022_Conflict_Resolution_in_Decision_Making_Teams_A_Longitudinal_Study

[9] Styles B, Torgerson C. Randomised controlled trials (RCTs) in education research –methodological debates, questions, challenges. Educ Res. 2018;60(3):255–264. doi: 10.1080/00131881.2018.1500194

[10] Braam A, Buljac-Samardzic M, Hilders CGJM, et al. Collaboration between physicians from different medical specialties in hospital settings: a systematic review. J Multidiscip Healthc. 2022;15:2277–2300. doi: 10.2147/JMDH.S376927 36237842 PMC9552793

[11] Kiesewetter J, Fischer F, Fischer MR. Collaborative clinical reasoning—A systematic review of empirical studies. J Contin Educ Health Prof. 2017;37(2):123–128. doi: 10.1097/CEH.0000000000000158 28562501

[12] Tschan F, Semmer NK, Gurtner A, et al. Explicit reasoning, confirmation bias, and illusory transactive memory: a simulation study of group medical decision making. Small Group Res. 2009;40(3):271–300. doi: 10.1177/1046496409332928

[13] Haidet P, Levine RE, Parmelee DX, et al. Perspective: guidelines for reporting team-based learning activities in the medical and health sciences education literature. Acad Med. 2012;87(3):292–299. doi: 10.1097/ACM.0b013e318244759e 22373620

[14] Burgess AW, McGregor DM, Mellis CM. Applying established guidelines to team-based learning programs in medical schools: a systematic review. Acad Med. 2014;89:678–688. doi: 10.1097/ACM.0000000000000162 24556770 PMC4885587

[15] Wiener H, Plass H, Marz R. Team-based learning in intensive course format for first-year medical students. Croat Med J. 2009;50(1):69–76. doi: 10.3325/cmj.2009.50.69 19260147 PMC2657558

[16] Michaelsen L, Richards B. COMMENTARY: drawing conclusions from the team-learning literature in health-sciences education: a commentary. Teach Learn Med. 2005;17(1):85–88. doi: 10.1207/s15328015tlm1701_15 15691820

[17] Palsolé S, Awalt C. Team-based learning in asynchronous online settings. New Dir Teach Learn. 2008;2008(116):87–95. doi: 10.1002/tl.336

[18] Clark MC, Merrick LC, Styron JL, et al. Orientation principles for online team-based learning courses. New Dir Teach Learn. 2021;2021(165):11–23. doi: 10.1002/tl.20433

[19] Anas S, Kyrou I, Rand-Weaver M, et al. The effect of online and in-person team-based learning (TBL) on undergraduate endocrinology teaching during COVID-19 pandemic. BMC Med Educ. 2022;22(1):120. doi: 10.1186/s12909-022-03173-5 35193577 PMC8863392

[20] Wyszomirska RM, Pennaforte RJ, de Barros Costa FG, et al. Team-based learning: a promising strategy for use in online distance education. Creative Educ. 2021;12(1):278–292. doi: 10.4236/ce.2021.121020

[21] Sannathimmappa MB, Nambiar V, Aravindakshan R, et al. Are online synchronous team-based-learning (TBL) pedagogy effective?: perspectives from a study on medical students in Oman. J Adv Med Educ Prof. 2022;10:12–21. 34981001 10.30476/JAMP.2021.92361.1481 PMC8720151

[22] Anon. Moving team-based learning online - faculty Guide. Available at n.d [cited 2023 Dec 13]. Available from: https://www.blog.intedashboard.com/guides/tbl-learning/online-tbl

[23] Ng M, Newpher TM. Class size and student performance in a team-based learning course. J Undergrad Neurosci Educ. 2021;20(1):A49–A57. 35540942 PMC9053426

[24] Sterpu I, Herling L, Nordquist J, et al. Team-based learning (TBL) in clinical disciplines for undergraduate medical students—a scoping review. BMC Med Educ. 2024;24(1):18. doi: 10.1186/s12909-023-04975-x 38172844 PMC10765894

