# Supplementary material for: Short-term cognitive learning outcomes in team-based learning: is the permanent team important?
Source: Med Educ Online. 2024 Sep 5;29(1):2397864. doi: 10.1080/10872981.2024.2397864 (PMC11382708; doi:10.1080/10872981.2024.2397864)
Supplement: Supplemental Material [file ZMEO_A_2397864_SM8282.docx]

# Supplementary Information

####
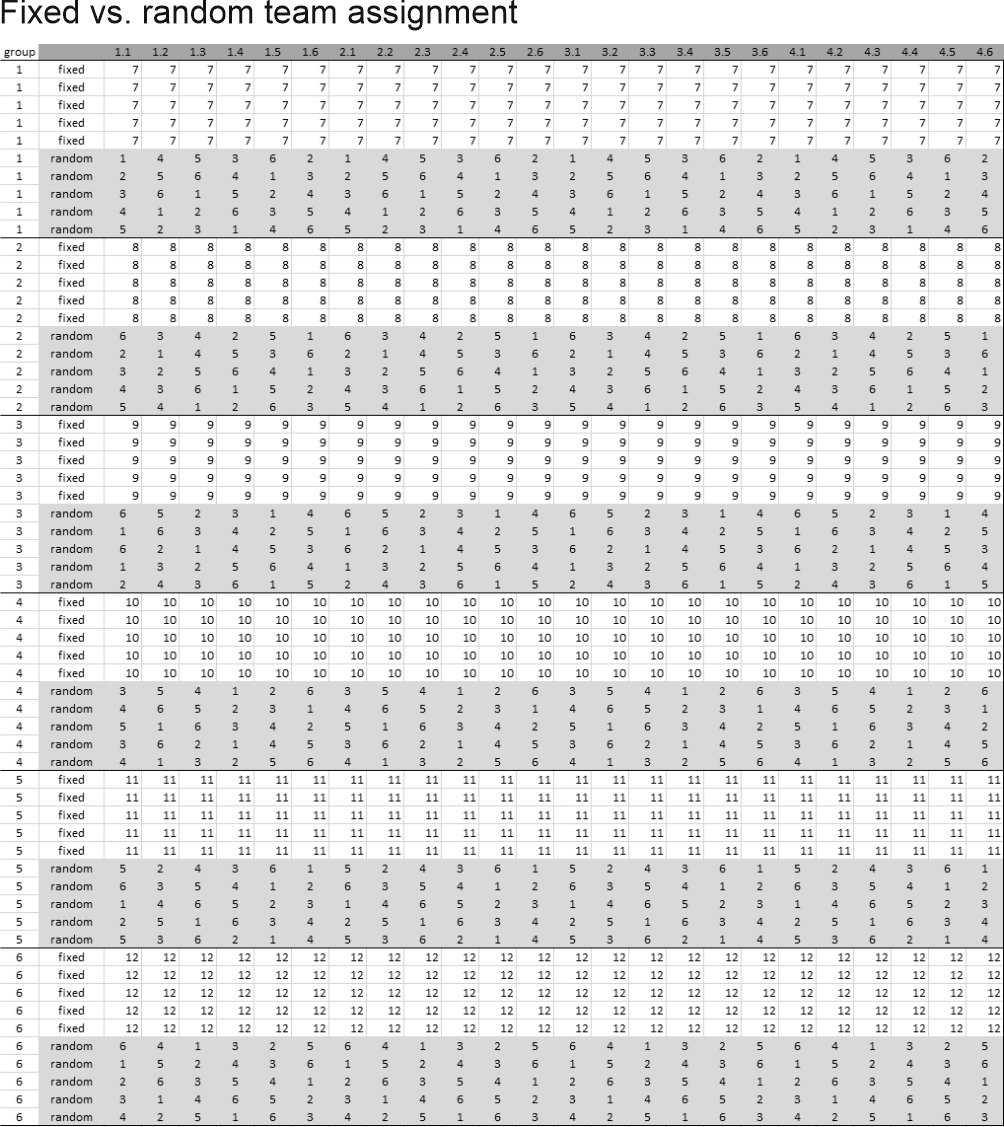


#### **Figure S1 - Predesigned team composition.** Left column indicates the university’s groups. Within a group, half was assigned to the permanent teams 7-12 (light background) and half to the temporary teams 1-6 (grey background). The top row indicates the assignment per online TBL and per problem, e.g. 2.3 refers to the third problem of the second online TBL. A similar table was generated for each online TBL, with an additional column containing the student's name, so that students could look up their team and enter the appropriate breakout room.

Preparation

- Start the online TBL room, e.g. via the link also provided to students in Moodle.
- Enable Voting Module (View→Panels→Manage Panels)
- Enable breakout sessions: Enable Breakout sessions*
- Load online TBL PPT online and 'share' it, briefly explain the timeline at first occasion, provide on slide 2

Individual voting 1-6, each (duration approx. 15 minutes)

- Show PPT problem
- Load problem via file (designated e.g. TBL2problem3.txt)
- Start voting (2 minutes are preset, can be terminated earlier)
- Save voting results
- DO NOT share poll results' with participants

Team phase, for each problem

- Show problem on PPT
- Start break-out rooms
- Broadcast problem on the slide to breakout rooms (PPTs are not visible, only student videos)
- Load problem via file (designated e.g. TBL2problem3.txt)
- Students return automatically within 5 minutes (see the countdown, so no announcement is required, they may return earlier), Break-out rooms can be terminated on request with a 60 s delay
- Start voting as soon as everyone is back (2 min, terminate when complete, assumed in less than one minute)
- Save voting
- Discuss all possible answers (5 min)

#### **Figure S2 - Short printable instructions for teachers, generated to support the first class**. A printed list of student names allowed teachers to mark teams and individuals with whom they had interacted. This helped to balance the interaction, which was otherwise difficult to achieve given the regular reassignment of students.

| **Core design elements of the TBL method** |  |
| --- | --- |
| immediate feedback | no feedback on iRATs  tRATs were shown to students in percentage of teams with a correct answer, students did not get to know the decision of a particular other team |
| sequencing of in-session problem solving | Intra-team discussion: Students were instructed to reach a consensus on a common solution.  Inter-team discussion: During the supervisor-led inter-team discussion teams were invited to explain their rationale for their decision  There was no pre-planned inter-team discussion, but this could have occurred through the teacher's moderation. |
| the four S’s  (significant problem,  same problem,  specific choice,  and simultaneous reporting) | All students worked on significant problems, designed to be challenging, as students should use their domain knowledge from all prior sessions on these topics. All problems and specific choices were identical for all students. Choices were electronically submitted within the given time, but progress of the polls was only visible to the teacher, and for tRATs the results were only made available to students after the end of the voting process. |
| incentive structure | No grades were given for iRAT and tRAT. Grading of the domain knowledge test was a separate curricular element |
| peer review | No formal peer-review procedure was implemented in the specific setting. |

**Table S1. Core design elements that underlie the TBL method.** Team composition and readiness assurance are not mentioned here, as these are described in text in detail.
